# Materials Based on Quaternized Polysulfones with Potential Applications in Biomedical Field: Structure–Properties Relationship

**DOI:** 10.3390/ijms23094721

**Published:** 2022-04-25

**Authors:** Alexandra Bargan, Mihaela Dorina Onofrei, Iuliana Stoica, Florica Doroftei, Simona Dunca, Anca Filimon

**Affiliations:** 1 Inorganic Polymers Department, “Petru Poni” Institute of Macromolecular Chemistry, Grigore Ghica Voda Alley 41A, 700487 Iasi, Romania; 2 Polycondensation and Thermostable Polymers Department, “Petru Poni” Institute of Macromolecular Chemistry, Grigore Ghica Voda Alley 41A, 700487 Iasi, Romania; mihaela.onofrei@icmpp.ro; 3 Physical Chemistry of Polymers Department, “Petru Poni” Institute of Macromolecular Chemistry, Grigore Ghica Voda Alley 41A, 700487 Iasi, Romania; stoica_iuliana@icmpp.ro; 4 Physics of Polymers and Polymeric Materials Department, “Petru Poni” Institute of Macromolecular Chemistry, Grigore Ghica Voda Alley 41A, 700487 Iasi, Romania; florica.doroftei@icmpp.ro; 5 Department of Microbiology, Biology Faculty, “Alexandru Ioan Cuza” University of Iasi, 11 Carol I Bvd., 700506 Iasi, Romania; sdunca@uaic.ro

**Keywords:** quaternized polysulfones, cellulose acetate phthalate, polyvinyl alcohol, electrospun fibers, surface properties, membrane permeability, antibacterial activity

## Abstract

Starting from the bactericidal properties of functionalized polysulfone (PSFQ) and due to its excellent biocompatibility, biodegradability, and performance in various field, cellulose acetate phthalate (CAP) and polyvinyl alcohol (PVA), as well as their blends (PSFQ/CAP and PSFQ/PVA), have been tested to evaluate their applicative potential in the biomedical field. In this context, because the polymer processing starts from the solution phase, in the first step, the rheological properties were followed in order to assess and control the structural parameters. The surface chemistry analysis, surface properties, and antimicrobial activity of the obtained materials were investigated in order to understand the relationship between the polymers’ structure–surface properties and organization form of materials (fibers and/or films), as important indicators for their future applications. Using the appropriate organization form of the polymers, the surface morphology and performance, including wettability and water permeation, were improved and controlled—these being the desired and needed properties for applications in the biomedical field. Additionally, after antimicrobial activity testing against different bacteria strains, the control of the inhibition mechanism for the analyzed microorganisms was highlighted, making it possible to choose the most efficient polymers/blends and, consequently, the efficiency as biomaterials in targeted applications.

## 1. Introduction

The polymeric biomaterials field includes a wide range of applications, as coatings in biomedical devices or as material in devices, such as catheters, heart valves, and dialysis membranes [1]. The polymers that were initially developed for a variety of industrial applications were subsequently adapted to be used as biomaterials, based on their specific characteristics, namely the wettability, chemical composition, porosity, flexibility/stiffness, roughness—properties significant in relation to the existing physiological environment [2,3]. Therefore, the development of new polymeric materials with sustainability, multifunctionality, and advanced properties is a challenge of our days. This research helps us to better understand the interaction nature from the systems and the relation between the structure, composition, and surface properties of the polymeric materials.

Most biological reactions occur on the surfaces and at the interfaces. For this reason, the performance of a material in biological environment is initially dependent on its surface properties and also bulk properties of the basic polymers. Therefore, the material surface, through its distribution and functionality and hydrophilic/hydrophobic balance [4], is the main factor that determines the biological response and, consequently, its biocompatibility [5]. In this context, polysulfones (PSFs) are known as high performance polymers with an excellent film-forming potential, great thermal stability, and an excellent resistance to inorganic bases and acids [6,7,8]. Besides these, other important and interesting properties of some types of PSFs, such as the transparency, hydrolytic stability, and resistance to heat, are relevant [5]. Due to its high hydrophobicity which causes an increase in the hydraulic resistance to water flow, a decrease in the water permeability and restrains the practical inclination in water treatment, there are some /researches in progress concentrated on changing the polysulfones in a way to provide a proper balance between hydrophobicity and hydrophilicity with the aim to make these materials suitable for needed applications. 

To meet these requirements, the structural modification of polymeric materials is essential. On the basis of the knowledge acquired from the structure–property relationships, a variety of polysulfones (PSFs) have been molecularly designed through the chloromethylation and quaternization reactions of PSFs [9,10,11,12]. Under these conditions, the functionalized PSFs membranes have not only advantages, e.g., antimicrobial, environmental, biocompatibility properties, but also disadvantages such as the fact that they behave as an obstacle in the separation process. This inconvenience can be surpassed by blending, thus improving in this way the water permeability and antifouling properties. Additionally, the added plasticizer improves the surface properties, the workability and membranes performance. Consequently, for an enhanced performance, design of blends/composites based on PSFs/functionalized PSFs using biologically inert and nontoxic compounds is preferred. The composite membranes consist of two materials with different characteristics: one of materials gives physico-chemical resistance, while the second one gives selectivity. In this context, the polysulfone-based composite membranes can be defined as an interphase limit-barrier which narrows the transport of various chemical species, in a selective way. From this perspective, in order to modify the inherent properties of PSFs/functionalized PSFs, conventional biodegradable polymers, such as cellulose and its derivatives (e.g., cellulose acetate phthalate (CAP)), polyvinyl alcohol (PVA), and polyesters have attracted attention and interest [13,14].

On the other hand, the surface modification has a relevant role in the biomedical applications due to the fact that the changes can help us to adjust the surface to the desired properties without making undesired changes in its bulk characteristics [15,16,17]. The correlation of these modifications (chemical and/or physical) assure the biocompatibility of PSFs and, implicitly, the possibility of their action in a living organism or in contact with living system without negative consequences [5].

In above-mentioned context, the literature indicates various studies concerning the synthesis and some properties of a series of quaternary ammonium-functionalized polysulfones [18,19,20,21]. Starting from the charged polysulfones with different ionic chlorine contents, their morphology and antimicrobial activity were evaluated [5]. The study object regarding their applications in membrane technology reveals that surface properties are influenced by the history of the processing protocols/techniques and by the charge density of the quaternized polysulfones. In addition, the adhesion of the microorganisms to the modified polysulfones solutions is determined in connection with the hydrophobic/hydrophilic characteristics of the polymers and tested microorganisms [22,23]. On the other hand, the systems based on functionalized polysulfones with tertiary amine have been obtained by mixing the solutions of functionalized polysulfone with a well-controlled content of different polymers [18]. These studies have led to development of an innovative way of designing the membrane materials with a long-term stable hydrophilicity, increased porosity, improved workability, and a very good biocompatibility. The obtained results represent the basis for future tests regarding their functionality as ionic-exchange membranes for hemodialysis [24]. 

In addition to combining the polymers properties by mixing, to induce a surface pattern with enhanced features, the processing protocols/techniques are very important. Thus, the nanomaterials obtained using the electrospinning technique possess a high surface area to volume ratio and large number of inter/intra-fibrous pores. The most relevant applications are drug delivery, tissue engineering, biosensors, filtration, wound dressing, and enzyme immobilization. The nanofibrous frames made by this method mimic the nanoscale properties of native extracellular components and can be adjusted according to their use. 

For this reason, unlike the previous studies, due to the bactericidal properties of functionalized polysulfone (PSFQ), the present study offers a development path of a new generation of composites, namely PSFQ/CAP and PSFQ/PVA blends/composites, projected as films and fibers to improve their performance, becoming biologically inert and nontoxic compounds, with stable mechanical, morphological and adhesion properties during their use. In this sense, due to their common characteristics, namely high hydrophilicity, flexibility, and good performances in film forming, which represent the important tools for improving and controlling the hydrophilicity and porosity of membranes, CAP and PVA were selected to be used in this study. Moreover, depending on their characteristics, it will be possible to choose the most efficient mixtures for uses in the biomedical field. Through this study, we follow the contribution/effect brought by each of the polymers added to the polysulfone matrix to improve the properties of the obtained active surfaces (membranes/films/fibers) designed to achieve multi-performance in bioinspired integrated system. 

## 2. Results and Discussion 

### 2.1. Surface Chemistry Analysis

**Figure 1:**
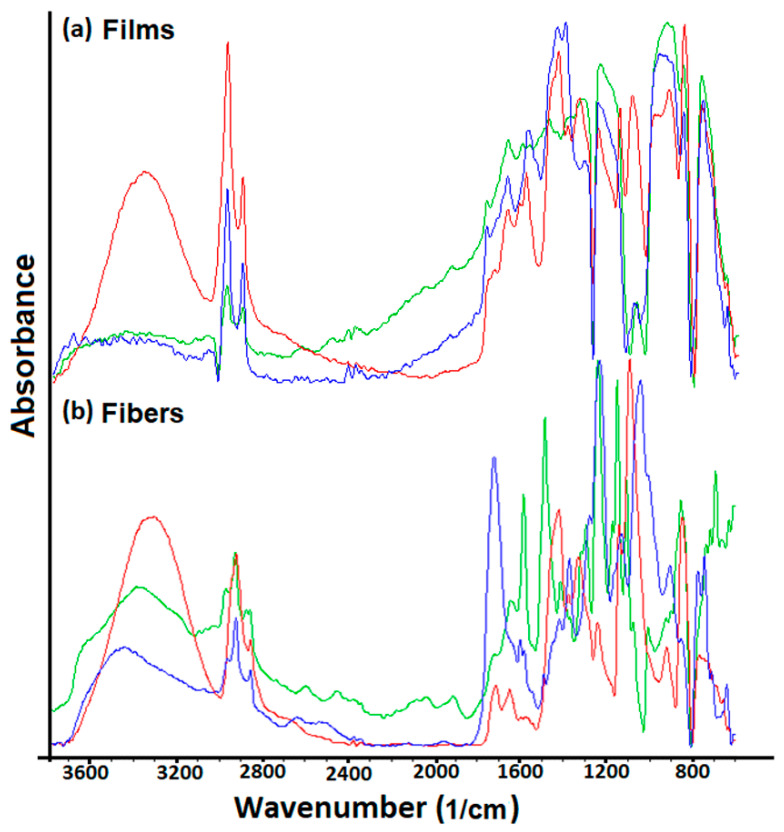
ATR-FTIR spectra of studied samples organized as films (**a**) and fibers (**b**) for PSFQ-green, CAP-blue, PVA-red.

The spectroscopic methods are key tools in determining the presence of the functional groups on the polymeric surfaces (Figure 1). Moreover, the inter- and intramolecular interactions due to the hydrogen bonding can be also determined through Fourier transform infrared spectra (FTIR) because these interactions affect the local electron density and a frequency shift could be observed [25]. 

Based on previous works [22,26], the FTIR spectra indicated the characteristic bands for the polymers studied. Thus, the FTIR spectrum for the PSFQ has presented the absorption bands, corresponding to -SO_2_ from the PSF backbone, around 1330 and 1300 cm^−1^ as asymmetric stretching, while the strong absorption band around 1150 cm^−1^ corresponds to the symmetric stretching. The aromatic structure was confirmed by the appearance of the absorption bands at approximately 1590–1410 cm^−1^ and those from 2990–2950 cm^−1^ and 2894–2850 cm^−1^ domains; bands attributed to the vibrations of aliphatic units (–CH_3_ and –CH_2_, respectively) were presented. The quaternary ammonium group was confirmed by the appearances of stretching vibration peak at approximately 1635 cm^−1^. On the other hand, in CAP spectrum, the following peaks were observed: around 2980 and 2883 cm^−1^, absorption bands are attributed to the asymmetric and symmetric stretching vibrations of methyl –C-H groups; at about 1750, 1725, and 1701 cm^−1^ the adsorption peaks are associated with the –C=O carbonyl group vibrations, while the –C=C– linkage of the aromatic ring showed a peak at about 1599 cm^−1^. At 1492 and 1284 cm^−1^ were found the characteristic bands of stretching vibrations of –C-H bond from methylene groups and –C-O-C– bond of the ester groups, respectively. Peaks from region 1140 to 1071 cm^−1^ were related to the –C-O– stretching vibrations of cyclic ether structure. Analysis of FTIR spectrum for PVA showed that the large band between 3500 and 3200 cm^−1^ was attributed to the O-H stretching vibrations and intermolecular hydrogen bonding vibration [14]. Two bands with maximum at 2940 and 2910 cm^−1^ are attributed to the asymmetric and symmetric alkyl C-H stretching vibrations. The absorption peaks at 1650 and 1570 cm^−1^ correspond to C=O stretching and C-O stretching of acetate group, and the peak from 834 cm^−1^ is related to the C–C stretching.

Generally, in the 3100–3500 cm^−1^ range, there is a wide absorption band attributed to free hydroxyl stretching vibrations (not hydrogen bonded) and to those that are self-associated (hydrogen bonded) with the hydroxyl groups [14,26]. The latter is more intense than that obtained for the free ones; however, the hydroxyl stretching may be affected by the presence of eventual hydrogen bonding interactions. Additionally, the FTIR carbonyl-hydroxyl stretching range or flexion mode hydroxyl–hydroxyl interactions are sensitive to the formation of new interactions between the polymer’s blends. In these conditions, when one of the polymers from the blend predominates, the appearance of a new band located at higher/lower wavelengths is identified, as a consequence of the inter- and intramolecular hydrogen bonding interactions between different species [14,26]. Therefore, the FTIR spectra shown in Figure 1 recorded over the 4000–600 cm^−1^ range include/highlight all these statements, identifying the different structuration types generated, on the one hand, by the orientation of the functional groups of the polymers during the processing processes and, on the other hand, by the occurrence of some interactions between different functional groups of the involved polymers in the formation of blends as a function of the organization form, film and/or fibers. All changes on the characteristic peaks revealed a good miscibility and interaction between components in the matrix due to the formation of the inter- and intramolecular hydrogen bonding.

Additionally, the evaluation of the intensity of characteristic vibration peak of functional groups recorded in ATR mode and their displacements to lower or higher values can demonstrate how the functional groups are distributed during the processing, casting solutions and/or electrospinning, and also how they are subsequently stabilized in the films/fibers structure. Moreover, in the case of a mixture of two polymers, the diffusion of a flexible polymer chain (e.g., CAP and/or PVA) into host matrix (PSFQ) is followed by the destruction of the weak bonds, changing the strength of some bonds among the atoms of the host polymer. Thus, other new bonds are created, and finally, the structural rearrangement of the entire macromolecular system takes place. 

In this context, the intensities of the peaks characteristic to the functional groups, as detected in ATR mode at wavenumber around of 1635 cm^−1^ (quaternary ammonium group for PSFQ), 3465 cm^−1^ and 1650–1750 cm^−1^, respectively (the hydroxyl groups and carbonyl stretching region, respectively, for CAP), and also the -OH absorption band for PVA at approximatively 3400 cm^−1^, are more pronounced in the case of polymers organized as fibers compared to those obtained for films, as a result of the functional groups orientation during electrospinning experiments. This tendency of changing in the films/fibers structure as a result of orientation/rearrangement of functional groups onto or inside of the surfaces is highlighted through the variation of peaks intensity.

Consequently, the evaluation and comparison of FTIR spectra recorded in ATR mode (ATR-FTIR) of the PSFQ, CAP, and PVA polymers in fiber and film form and their blends are needed to offer the right views on the behavior of the materials and performances of the membranes and fibers, respectively, for future applications in biomedical domain.

### 2.2. Rheological and Wetting Parameters

**Figure 2:**
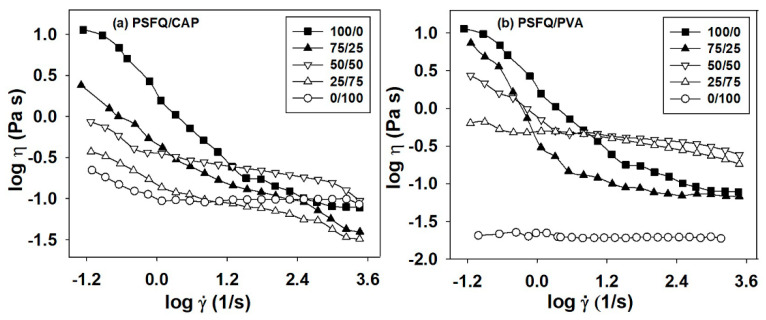
Double–logarithmic plots of dynamic viscosity (η) versus shear rate ($\dot{\gamma }$) for: (**a**) PSFQ/CAP and (**b**) PSFQ/PVA blends at various mixing ratios (wt./wt.) at 25 °C.

Rheological behavior of the polymer solutions plays an important role in defining the properties of the final products with applicability in certain areas, influencing the obtaining process of the films/fibers and, consequently, their surface properties. In this context, the rheological investigations of pure components (PSFQ, PVA, and CAP solutions in N-methyl-2-pyrrolidone, NMP) and of their blends, [18,26] in terms of the dynamic viscosity–shear rate dependence, where complex behavior appears as result of the polymers properties and blends composition that were previous analyzed (Figure 2). Thus, the investigation of flow curves evidenced a shear thinning behavior at low shear rate, followed by a linear domain where the viscosity is constant (Newtonian plateau), when shear rate increases.

These results indicate significant changes in the PSFQ/CAP and PSFQ/PVA systems as result of the specific molecular rearrangement of the polymeric chains in blend, determined by the plasticizer effect of CAP and PVA in the PSFQ solution. Moreover, according to the literature studies on the mechanism of shear thinning [27,28,29], the shear flow could reduce the probability of interchain association at the expense of intrachain association, thus leading to a lower viscosity (Figure 2a). This behavior is due to the higher orientation of the polymer chains of the CAP compared to those of PVA in the PSFQ solution. Therefore, we can conclude that these blends possess properties of pseudoplastic materials, characterized by reduced entanglement density and enhanced number of the oriented segments, as result of shear rates increasing. 

**Figure 3:**
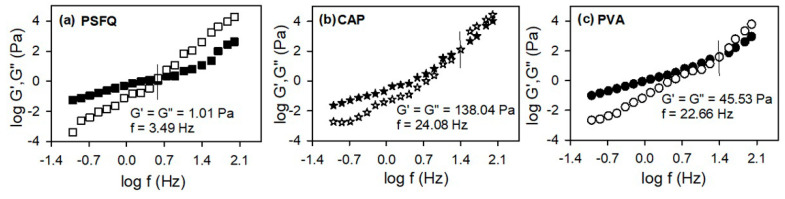
Double–logarithmic plots of shear moduli (*G*′ and *G*′′) versus oscillatory frequency (f) for pure sample: (**a**) PSFQ ($G^{\prime }$ (□),$G^{\prime \prime }$ (■)); (**b**) CAP ($G^{\prime }$ (✩),$G^{\prime \prime }$ (★)); (**c**) PVA ($G^{\prime }$ (○), $G^{\prime \prime }$ (●)) at 25 °C.

**Figure 4:**
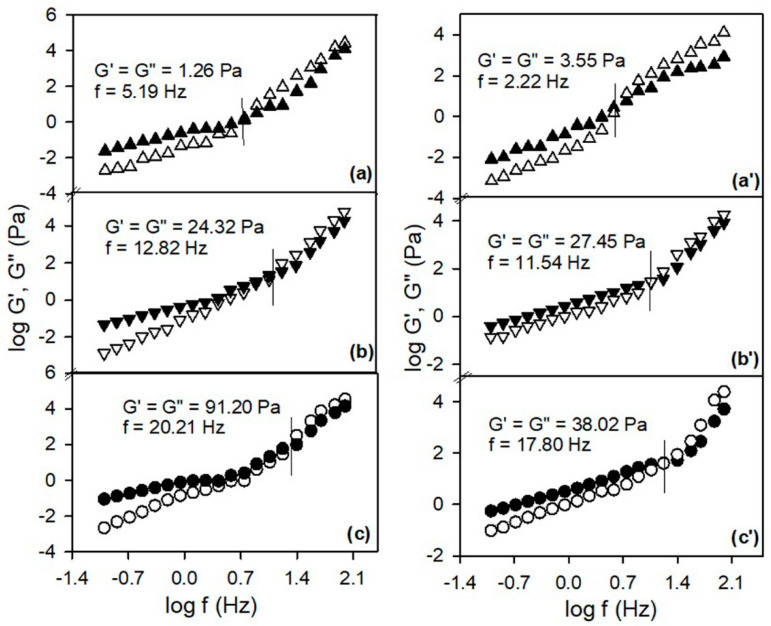
Double–logarithmic plots of shear moduli versus oscillatory frequency (f) at various mixing ratios (wt./wt.) for PSFQ/CAP (**a**–**c**) and PSFQ/PVA (**a’**–**c’**) blends at 25 °C. (**a**,**a’**) 75/25 ($G^{\prime }$ (∆), $G^{\prime \prime }$ (▲)); (**b**,**b’**) 50/50 ($G^{\prime }$ (▽), $G^{\prime \prime }$ (▼)); (**c**,**c’**) 25/75 ($G^{\prime }$ (○), $G^{\prime \prime }$ (●)).

The above-mentioned findings concerning the influence of the structural characteristics of PSFQ, PVA, CAP and variation of the composition of the polymer blends on the chains mobility in the shear field was also confirmed by evaluation/analysis of the storage, $G^{\prime }$, and loss, $G^{\prime \prime }$, moduli (Figure 3 and Figure 4). Thus, the rheological response for PSFQ, CAP, PVA pure polymers and their blends, at different compositions, over the low oscillatory frequency range exhibited a characteristic behavior typical for the viscous liquid, where $G^{\prime \prime }>G^{\prime }$.

In addition, the overlap frequencies that delimit the viscous flow from the elastic one, occur at high frequencies, reflecting the influence of polymer chain flexibility and arranging the polymers in following order: PSFQ < PVA < CAP (Figure 3). Therefore, the rheological behavior of PSFQ/PVA and PSFQ/CAP blends is influenced by specific interactions, like hydrogen bonding and association phenomena from the complex systems. In conclusion, as a result of the shear rates and overlap frequencies increasing, the rheological data were reflected in morphological changes induced by the PVA or CAP presence in PSFQ solution.

Successful application of a polymeric material in a biological environment is determined by its bulk and surface properties and is also is mediated by the combination of the solution and surface specific characteristics that are required for specific application [4,30]. These combined studies contribute to a better knowledge of the system interactions nature, and of the relation between the shear deformation and texture. Therefore, the surface of the biomaterial represents a key factor, determining the biological response and, therefore, the biocompatibility [31]. Thus, properties such as the flexibility/stiffness, wettability, porosity, roughness of the biomaterial’s surface, are highly significant with respect to the existing physiological environment [2]. 

It is known that the flexibility of polymers is closely related with their hydrophilicity, so besides the rheological studies, in order to design blends based on quaternized polysulfones as biomaterials, the interpretation of their surface characteristics and control of physical or/and chemical modificationsmust be taken into account. Generally, their biocompatibility is mainly affected by the surface characteristics, including hydrophilic/hydrophobic balance of the surface, distribution and functionality, and surface smoothness [32]. Hence, the evaluation of the hydrophobic/hydrophilic balance is essential for studying the compounds used as biomaterials with different applications in biomedicine and biotechnology. In particular, as is shown in the rheological studies, the modification of surface functional groups—as a result of change in the polymer blends composition and polymers nature from the casting solutions (PSFQ/CAP and PSFQ/PVA)—leads to an increase in flexibility and workability of the films. Therefore, this modification plays an important role in biomedical applications because the changes can be used to adapt the surface to the desired characteristics without compromising its bulk properties. Thus, the effect of the different functional groups on the surface properties was evaluated from water contact angle measurements ($\theta_{water}$) by surface free energy, $\Delta G_{w}$, (Equation (1) using the total surface tension of water, $\gamma_{lv}$= 72.80 mN/m [33])) which expresses the balance between the surface hydrophobicity and hydrophilicity.

(1) $$\Delta G_{w}=-\gamma_{lv}(1+\cos\theta_{water})$$

**Figure 5:**
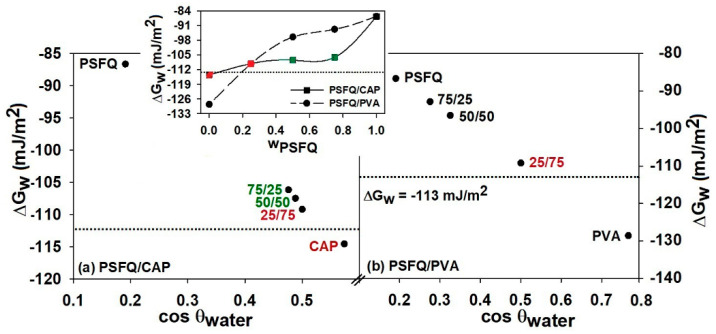
Surface free energy evaluated from contact angle measurements for the films of the studied systems: (**a**) PSFQ/CAP and (**b**) PSFQ/PVA. The small inserted graph represents the variation of surface free energy as function of the systems composition.

As is known, the water contact angle () is an indicator of the wettability of the film surface [5,33], and the approach of the polymer surface energy allows us to correlate the surface tension parameters ($\gamma_{lv}$) with their chemical nature. Thus, the hydrophobic surfaces, with high contact angles, were converted into hydrophilic surfaces, with low contact angles, through the CAP or/and PVA addition in PSFQ matrix (Figure 5). Thus, this versatility, in function of the final application requirements, is mainly attributed to the presence of a great number of functional groups (e.g., hydroxyl, carbonyl) which confer to PSFQ chain a high flexibility and hydrophilicity. 

According to the literature, a polymer surface can be considered more hydrophilic for $\Delta G_{w}<-113$ mJ/m^2^, whereas, when $\Delta G_{w}>-113$ mJ/m^2^, it should be considered more hydrophobic. Taking into account these statements, the polymers’ nature and modification of the composition in the studied systems (PSFQ/CAP and PSFQ/PVA) determine a decline in the contact angles and an appropriate increase in their surface wettability compared with the PSFQ sample, caused by the addition of polar groups to the PSFQ matrix, which exhibit high hydrophilicity (see Figure 5). The modification of the wetting characteristics of the studied surfaces can be caused by the polymer chains orientation and rearrangement of the polar groups on the film surface, after the blending process, due to the increase in the local polar moments. Moreover, analyzing the data obtained for the two studied systems, it can be stated that in the case of the PSFQ/CAP system due to the carbonyl and hydroxyl groups which increase the polarity, the surfaces have an improved hydrophilicity compared to PVA. However, we can conclude that the negative values of $\Delta G_{w}$ reveal an increased wettability, implicitly a high hydrophilicity—a property useful for biomedical applications—of the films obtained from solutions based on PSFQ- blends.

### 2.3. Surface Morphology Analysis

As it was observed from the study in solution, the conformational modifications of PSFQ chains in polymers blends, generated by specific interactions are reflected in the topographic organization of the polysulfonic films induced by the nature and content of the added polymers. Therefore, supported by literature [4,22,30], our results reported that the chain shape of a polymer in solution could affect the polymer morphology in bulk. Thus, in order to understand the effects induced by the presence of CAP or/and PVA in the polysulfonic matrix, a comparative analysis on the film surface aspects for PSFQ, PVA, CAP pure polymers and their blends is necessary. The addition CAP or PVA—which are flexible and hydrophilic polymers, namely to the polysulfonic matrix as plasticizers—improves the material properties by the interactions that it generates, such as intermolecular hydrogen bonds, and, consequently, changes the structure of the polymer both at molecular and morphological level.

**Figure 6:**
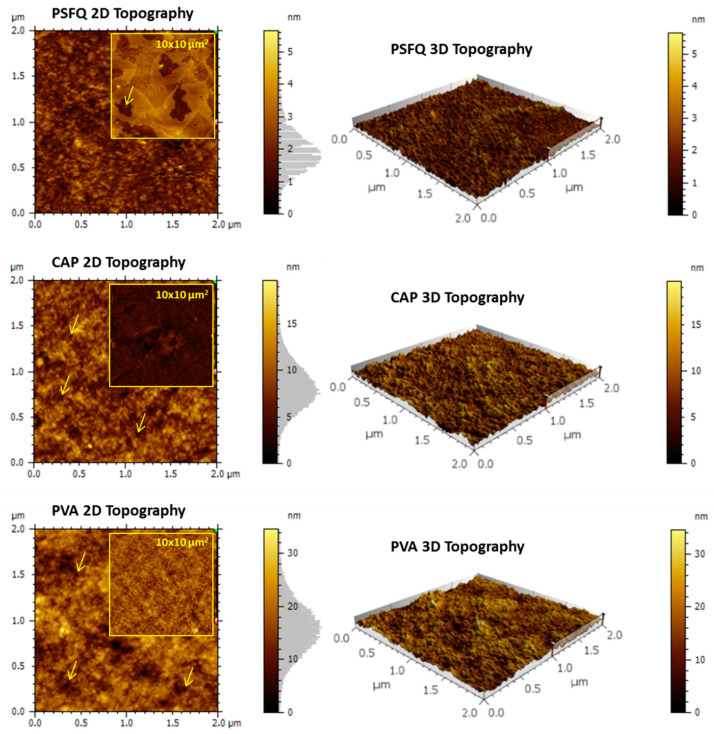
2D and 3D AFM topography images of the pristine polymers: PSFQ, CAP, and PVA.

**Figure 7:**
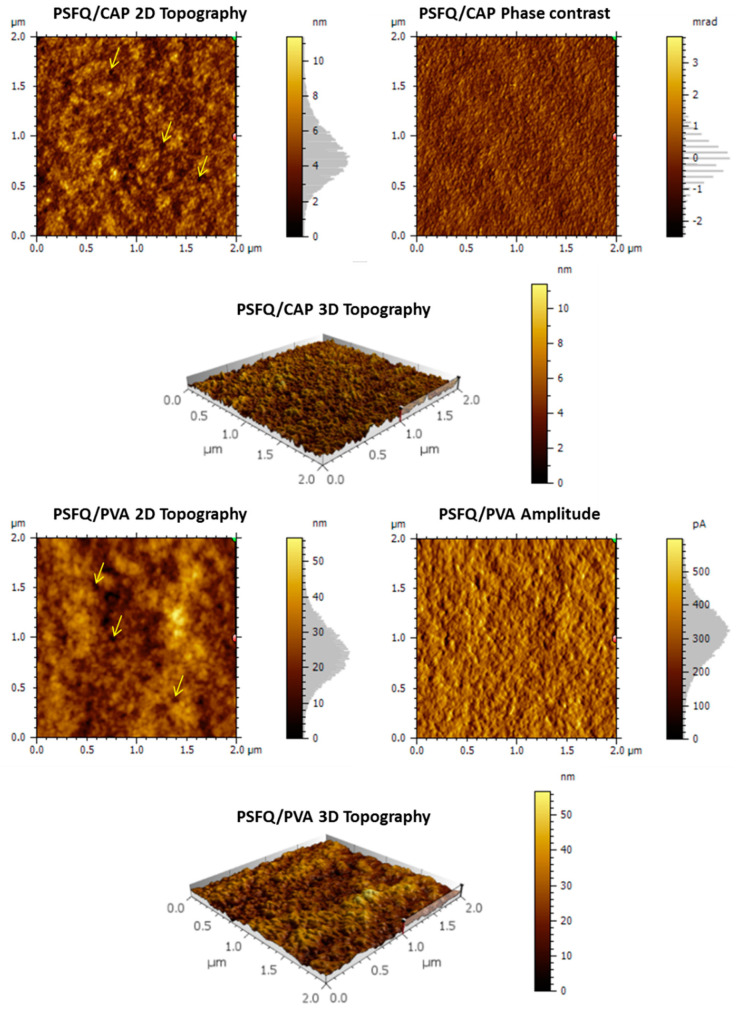
2D and 3D AFM topography images and phase contrast/amplitude images of the PSFQ/CAP and PSFQ/PVA blends at 25/75 mixing ratios (wt./wt.).

The morphology dictated by the roughness and the existence of porous formations are determining factors in order to obtain high-performance surfaces used in the biomedicine. In this context, atomic force microscopy (AFM) investigations of the films obtained from PSFQ- based blends can clarify the polymer influence, namely CAP or/and PVA, on the morphological properties, as well as its ability to improve the film-forming characteristics (Figure 6 and Figure 7). In this context, according to behavior evidenced by rheological measurements, the CAP or/and PVA added in different amounts acts as a plasticizer, generating an increase in the flexibility/workability of the films. Moreover, in accordance with data obtained through contact angle measurements was found that the addition of a large amount of CAP or/and PVA to PSFQ matrix (compositions containing less than 50 wt.% PSFQ in the blends) has conducted an increase in surface hydrophilicity. Therefore, the expected effect, to introduce modifications at the nanoscale in PSFQ matrix, is given by the structural and compositional characteristics of the other two polymers (CAP and PVA) used as hydrophilic additives and pore-forming agents. For this reason, the chosen films for further investigations were those containing high content of CAP or PVA, namely 25/75 wt./wt. compositions of the PSFQ/CAP or PSFQ/PVA blends, respectively (Figure 7). On the other hand, considering the excellent characteristics of CAP (e.g., a high chemical reactivity, the flexible structure of the polymer chain, and high hydrophilicity due to hydroxyl, carbonyl groups, etc.), the blends based on PSFQ—PSFQ/CAP—would bring new benefits to those already known from the literature, leading to materials with improved properties suitable for targeted applications.

Analysing the texture of the samples recorded by 3D and 2D AFM images, different morphologies are observed (Figure 6 and Figure 7) highlighting that the nature and composition of the polymers incorporated in the PSFQ matrix (CAP and/or PVA) are responsible for the formation of areas with pores and grains of different sizes and densities. In accordance with the above mentioned, the AFM image recorded for PSFQ (Figure 6) indicates the appearance of irregular, porous formations, randomly disposed in some areas, (the average diameter of these pores being of 2.16 ± 0.28 μm). Instead, in the case of CAP and PVA samples (Figure 6), the AFM images show a well-balanced surface relief with a nanoporous structure aspect (hardly observable on the 10 × 10 μm^2^ areas—inserted images). This change is in accordance with the previous statements regarding the flexibility and hydrophilicity of the samples. The average diameter of the nanopores detected and calculated using the Pore Analysis Module was 56 ± 17 nm for the CAP sample and 78 ± 15 nm for the PVA sample. Some of them are evidenced using arrows in Figure 6. 

On the other hand, when the PSFQ matrix is modified with a certain amount of plasticizer (i.e., 0.75 weight fractions of CAP or PVA), the surface of films obtained from PSFQ/CAP or PSFQ/PVA blends, respectively, become more complex (see Figure 7). Increasing the CAP or PVA content generates an increase in the number of nanopores but with different characteristics, namely the sizes being of 63 ± 12 nm for 25/75 wt./wt. composition of PSFQ/CAP blend compared to 101 ± 21 nm for 25/75 wt./wt. composition of PSFQ/PVA blend. This changing trend in film morphology coincides with the shear thinning behavior, evidenced from rheological data, as an effect of the conformation modification of polymeric chains in solution. The phase contrast and amplitude images presented in Figure 7 indicate that there is no phase contrast (phase separation) is presented at this scale, the phase shift/amplitude shift being due only to the differences in the surface relief heights. Moreover, the microstructural organization of the studied films is related to the surfaces wettability property as result of the chains orientation effect and polar group’s rearrangement on the films surface. 

**Figure 8:**
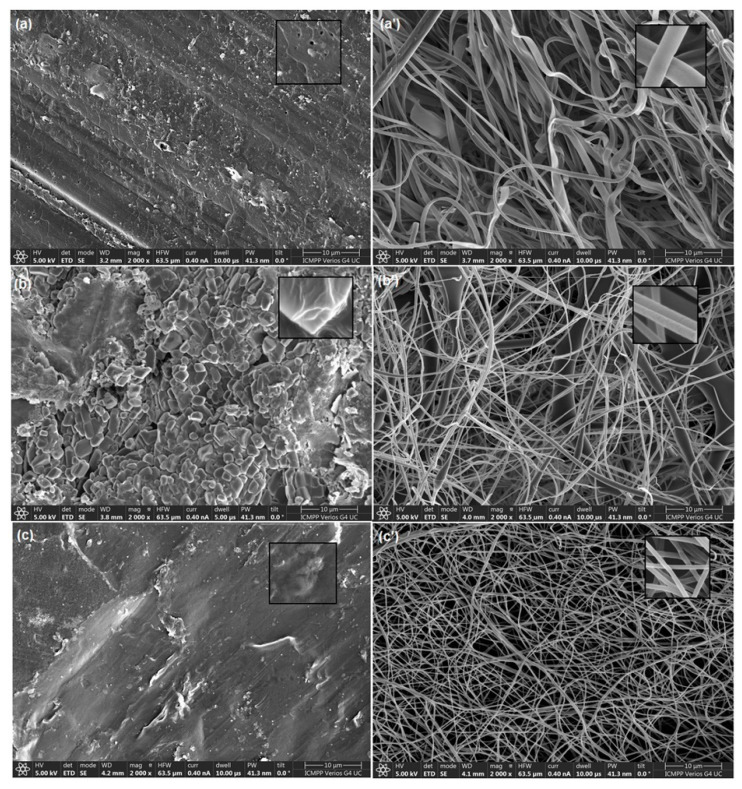
SEM topography images of the pure samples organized in the form of films and fibers, respectively: PSFQ (**a**,**a’**), CAP (**b**,**b’**) and PVA (**c**,**c’**) recorded for 10 µm scan area. Small images obtained at 2 µm were inserted for a better visualization.

The same changes in the texture and morphology of the polymer films can be seen also in the SEM images (Figure 8a–c). Thus, the SEM images for the PSFQ sample (Figure 8a) show a material with pores at the surface of the polymer, whose walls are more contoured. The SEM image for pure PVA film (Figure 8c) displays a more compact structure, which can confirm the presence of a homogenous surface due to the polymer characteristics, with no clearly visible pores. Instead, SEM images for CAP film (Figure 8b) show a homogenous surface with layered microparticles in the form of rectangles with variable sides between 0.5 and 4 microns, with well-defined roughness as wavy nano ripples (20–50 nm average roughness) developed on the surface of each microparticle. 

On the other hand, the electrospinning of PSFQ, CAP, and PVA solutions gave continuous cylindrical and thin fibers that were defect-free and randomly-oriented with inter-related voids among the fibers (Figure 8a’–c’). The examination of SEM micrographs revealed that PSFQ fiber (Figure 8a’) present loose smooth fibers with silky ribbon-like shape, with the width around 1 micron and average length over 150 microns. The surface of the PSFQ fibers is homogeneous with no roughness. Meanwhile, the CAP fiber (Figure 8b’) consists of loose fibers with overall tubular structure with the fiber radius of around 200 nm and average fiber length over 50 microns. The surface of the fibers shows roughness with wave-like ripples perpendicular to the fiber axis. As can be seen in the Figure 8c’, the PVA fiber consists in close-layered fibers with the fiber radius of around 200 nm and average fiber length over 100 microns. The surface of the fibers shows homogeneous aspect with no roughness. The differences between the morphology and polymers form have a relevant contribution to their behavior as biomaterials. 

Following the studies performed, we can conclude that the components are stabilized in the structure of the obtained films and fibers and also considering the superior properties acquired, namely improved flexibility, hydrophilicity, and specific molecular microarchitecture—properties required by biomedical applications. Studied materials can be recommended as surfaces/porous scaffolds with expected future developments in the biomedical fields.

### 2.4. Water Uptake Ability and Water Vapour Sorption Behavior

**Figure 9:**
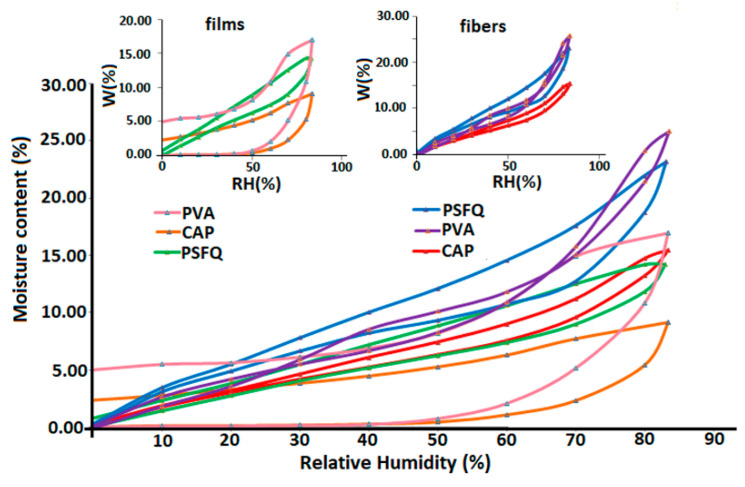
Sorption–desorption isotherms for studied polymers, in form of film and fiber. Small graphs have been inserted for better visualization depending on the processing mode. Registration conditions: equilibrium time 30/60 min.

**Figure 10:**
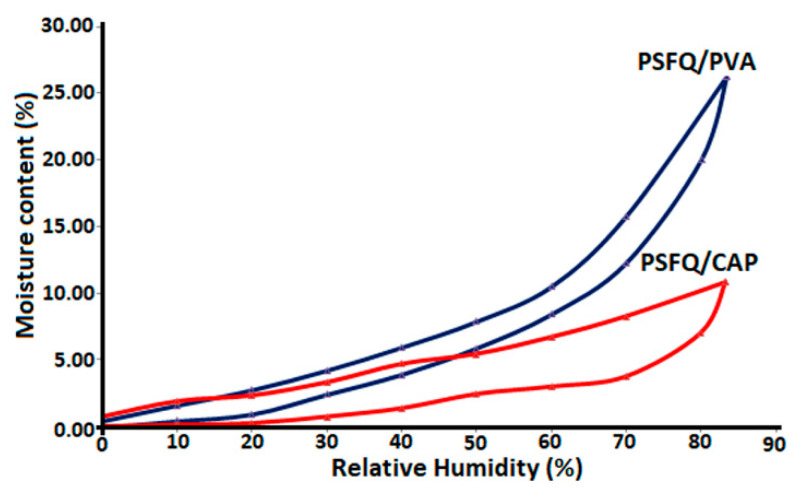
Sorption-desorption isotherms for the PSFQ/CAP and PSFQ/PVA studied blends at 25/75 mixing ratios (wt./wt.).

Adsorption isotherms are relevant for modeling, simulating, and optimizing of the studied systems in practical applications. In this context, considering the shape of the water sorption curves presented in Figure 9 and Figure 10, these can be associated with isotherms of Type IV, according to the IUPAC classification. This type of isotherm with hysteresis can be interpreted as being specific to the porous surfaces, providing information about the samples surface. Therefore, the following occurs: a reduced water vapor sorption capacity at low values of relative humidity, RH (0–10%), moderate sorption at intermediate values of RH, and a high increase of water vapour sorption at RH values close to 100%. The values of the surface parameters determined from adsorption/desorption isotherms for all studied samples are summarized in Table 1. 

The samples required an increased equilibrium time (30/60 min) for correct registration. The equilibrium is becoming more difficult to reach, especially for higher humidities, due to the possible cleavage of the physical bonds in the sample. In this condition, the surface characteristic properties are given by the sigmoidal shape and the presence of the hysteresis. They are reflecting adsorption via mono-multilayer (mainly multilayer) and capillary condensation in pores. The hysteresis loops and water vapour uptake levels are strongly influenced by the hydrophobic/hydrophilic balance modification. Moreover, the swelling and water vapour sorption behavior depend on material structure (composition, water affinity related to the presence of polar groups), surface area, morphology (topography and porosity, pores sizes and shape). The pores sizes and shapes, as well as the presence of polar groups with water affinity, are important in moisture sorption behavior of the samples. Thus, there are not high differences between the porous, macroporous and dense 3D structures (Figure 9 and Figure 10). 

To appreciate the specific surface area (Table 1), Brunauer–Emmett–Teller kinetic model (BET, Equation (2)) was applied by modeling the sorption isotherms registered under dynamic conditions.

(2) $$W=\frac{W_{m}CRH}{\left(1-RH)(1-RH+CRH\right)}$$

where the parameters involved are as follows: weight of sorbed water—W; weight of water forming a monolayer—$W_{m}$; sorption constant—C, relative humidity—RH.

The BET model describes the sorption isotherms up to a relative humidity of 40%, depending on the type of sorption isotherm and on the type of material. BET model can characterize the isotherms of types II but also types I, III and IV. The nature of the functional groups of the polymers from the system influence the values of water vapour capacities and, as a consequence of this fact, influence also the specific surface area and the average pore size of the studied samples.

By applying the Barrett, Joyner and Halenda model (BJF, Equation (3)), based on calculation methods for cylindrical pores, the average pore size, $r_{pm}$, (Table 1) was determined using the desorption branch of the isotherm. The desorbed amount of vapor is due either to the evaporation of the liquid core or to desorption of a multilayer. Pore size distribution is outlined as the distribution of pore volume. The relationship between pore volume and moisture uptake can be defined knowing the density of the adsorbed phase. The first assumption of mesopore size analysis is that this phase is equivalent to the liquid phase of the adsorbate.

(3) $$r_{pm}=\frac{2W}{100\rho_{a}A}$$

where W is absorption percentage, $\rho_{a}$ is the adsorbed phase density, and A is specific surface area evaluated by BET method. 

Investigations on studied surface, based on the water vapor sorption data, have demonstrated that on their surface exist nanosized pores whose number varies from sample to sample, as a function of the structural peculiarity of polymers and the number of polymers in the blend. It is notable the porogen effect of the CAP and PVA which generates structures with a high specific surface area and small pore size into the polysulfone matrix. That is why it is necessary to provide a high pore density and, consequently, a high permeability. On the other hand, analyzing the results, values of the water vapors’ sorption capacities and surface characteristics for studied fibers increase compared to the ones corresponding to the films. This tendency of changing as a result of orientation/rearrangement functional groups (highlighted by ATR-FTIR data) is consistent with the topographical reorganization of the films/fibers, revealing that the specific molecular rearrangements produced in the system through modification of the mixing ratio of polymers influence the surface properties. Consequently, it can be stated that the surface properties of the materials are those that dictate their functionality, performance and implicitly their biological ability, and not their specific surface.

### 2.5. Testing the Antibacterial Capacity 

One of the important issues in the medical field is the obtaining of the porous scaffolds with specific physical and biological properties. In this context, materials used for applications in this domain must be designed to stimulate the specific cell response at the molecular level, inducing specific attachment, proliferation, and differentiation of cells. In addition to cell–material interaction, the antibacterial properties also play an important role in the medical field. From this perspective, the selection of the biomaterials constitutes the most important factor for the success of practical applications in health and medicine field. Thus, it is well known that some positively charged polymer materials present antibacterial properties because they kill the bacteria by disrupting the negatively charged cell bacteria membrane (containing phosphatidylethanolamine as the major component) as result a of their charge. In this regard, the functionalized polysulfones with quaternary ammonium groups (PSFQ) are the most explored kind of polymeric biocide that offers some advantages including good antibacterial activity, reactivity, films and fibers forming capacity, and favorable hydrophilicity [26,34].

**Figure 11:**
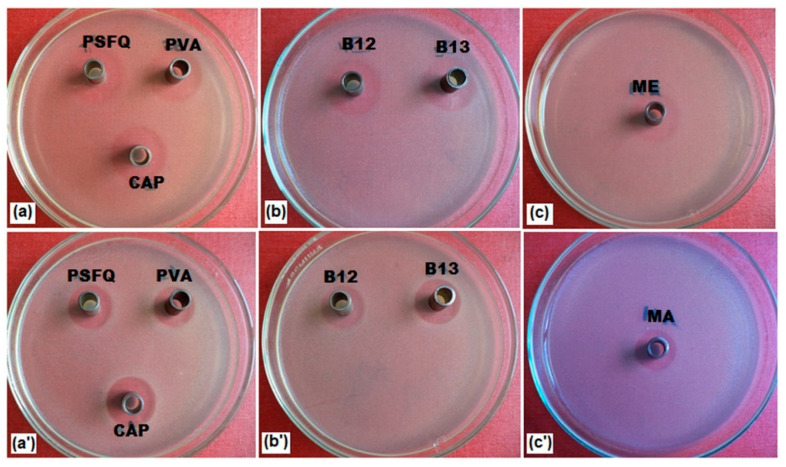
Testing the antibacterial activity of studied materials and their blends (70/30 wt./wt. composition of PSFQ/PVA and PSFQ/CAP blends, noted B12 and B13, respectively) at concentration of 0.5 g/dL against *E. coli* (**a**,**b**) and *S. aureus* (**a’**,**b’**)). In figure was presented images recorded for NMP used as a control sample (noted ME (**c**) and MA (**c’**)).

On the other hand, although a single-component polymer material cannot satisfy all requirements, the design and preparation of the multicomponent polymer systems constitute a viable strategy, assuring the progress in the multifunctional biomaterials domain. Therefore, the modification procedures increase hydrophilicity and porosity of surface, having an important effect on the flux and fouling reduction, improving the antibacterial activity and blood compatibility. In this context, the use of polymeric materials with antimicrobial properties, namely PSFQ, CAP and PVA, gains an increasing interest from both academic and medical perspectives. In particular, the antibacterial activity of PSFQ, CAP and PVA, and their blends (70/30 wt./wt. composition of PSFQ/CAP and PSFQ/PVA blends) using *Escherichia coli* (*E. coli*) and *Staphylococcus aureus* (*S. aureus*) microorganisms in terms of the inhibition zone diameter, was evaluated (Figure 11). The obtained results indicate the presence of well-defined inhibitory zones, the aspect which demonstrates that these materials have antibacterial activity against tested microorganisms.

Knowing from the literature the nature of the tested microorganisms as a result of the cell walls components, namely hydrophilic *E. coli* and hydrophobic *S. aureus* [35], we found that *E. coli* is more sensitive to the investigated materials than *S. aureus*. On the other hand, it should be noted that the inhibition effect of the tested samples on the *S. aureus* and *E. coli* microorganisms are slightly different because all polymers present hydrophilic character. Thus, considering the hydrophilic character of the studied samples (hydrophilicity of tested samples varying in the order of PSFQ < PVA < CAP), the inhibition of *E. coli* to PSFQ is slightly pronounced comparatively with the inhibition for PVA and CAP (values of the inhibition zone diameter being 23 mm for PSFQ > 22 mm for PVA > 20 mm for CAP). Moreover, the analysis of the inhibition zone diameters measured against to *E. coli* and *S. aureus* for studied blends (70/30 wt./wt. composition of PSFQ/CAP and PSFQ/PVA blends) indicates that the character of pure polymers gives the blends the same variation on antimicrobial activity.

Additionally, we mention that the obtained results are consistent with the experimental data previously presented [26] for the PSFQ and CAP samples at concentrations varying between 0.5 and 2 g/dL, maintaining the same inhibition trend. Consequently, antibacterial efficacy of the studied materials, using *Staphylococcus aureus* and *Escherichia coli* microorganisms, was demonstrated, and the obtained data indicate the potential for their application in health and medicine fields. 

## 3. Experimental Section

### 3.1. Materials 

**Scheme 1:**
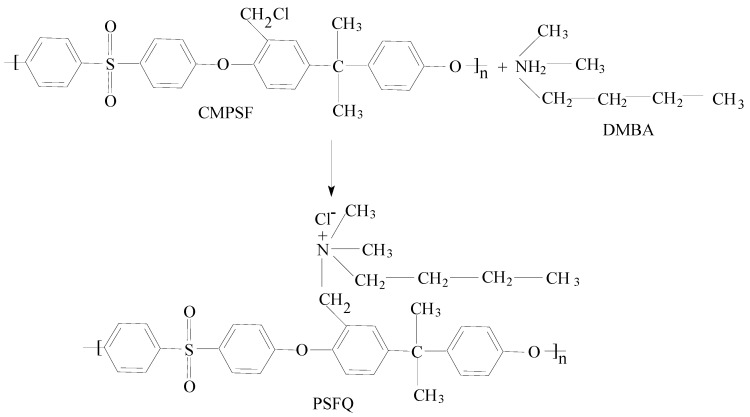
Obtaining quaternized polysulfone (PSFQ) by reaction of the chloromethylated polysulfone (CMPSF) with N,N-dimethylbutylamine (DMBA).

The functionalized polysulfone containing quaternary ammonium side groups (PSFQ, ${\bar{M}}_{n}=28,000$ g/mol, polydispersity index (PDI) of 2.64) was obtained by the substitution of the chlorine atoms of the chloromethylated polysulfone (CMPSF with a content in chlorine of 7.42% and ${\bar{M}}_{n}=29,000$ g/mol, PDI = 2.31) with ammonium groups using N,N-dimethylbutylamine as a functionalization agent [10] (Scheme 1). In previous studies [18,20], the detailed procedure of reactions for obtaining the PSFQ was presented. 

**Scheme 2:**
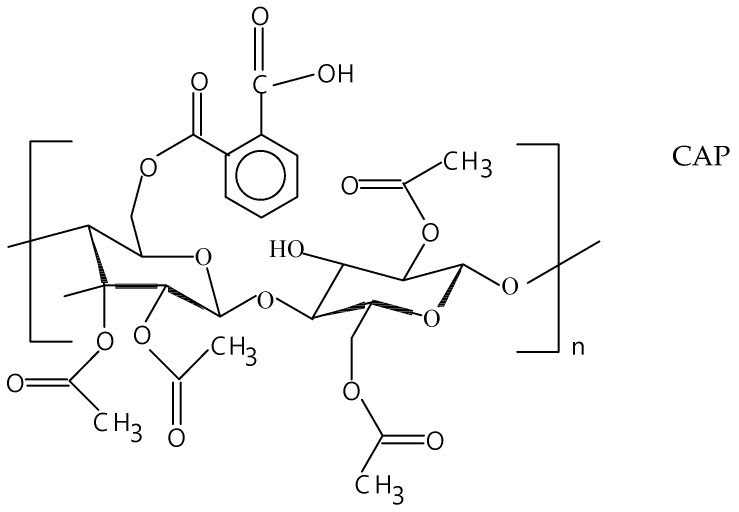

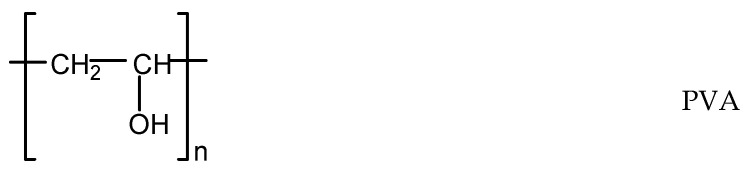
General chemical structures of cellulose acetate phthalate (CAP) and polyvinyl alcohol (PVA).

Cellulose acetate phthalate (CAP, ${\bar{M}}_{n}=2534$ g/mol, PDI = 1.088, high purity ≥ 99.5%, Sigma Aldrich, St. Louis, MO, USA) and polyvinyl alcohol (PVA, ${\bar{M}}_{w}=23,000$ g/mol, PDI = 1.094, hydrolysis degree around 98.8%, high purity powder, Celanese Corporation, Texas) were used as hydrophilic additives and pore forming for improving the characteristics of the films/fibers. The general chemical structures of CAP and PVA are illustrated in Scheme 2.

N-methyl-2-pyrrolidone (NMP, stated purity of 99.5%, Sigma Aldrich, St. Louis, MO, USA) was used as solvent.

The Gram-positive *Staphylococcus aureus* (ATCC 25923) and Gram-negative *Escherichia coli* (ATCC 25922) were used as test microorganisms. 

### 3.2. Processing of PSFQ Solutions 

#### 3.2.1. Preparation of Films by Solution-Casting Method 

**Figure 12:**
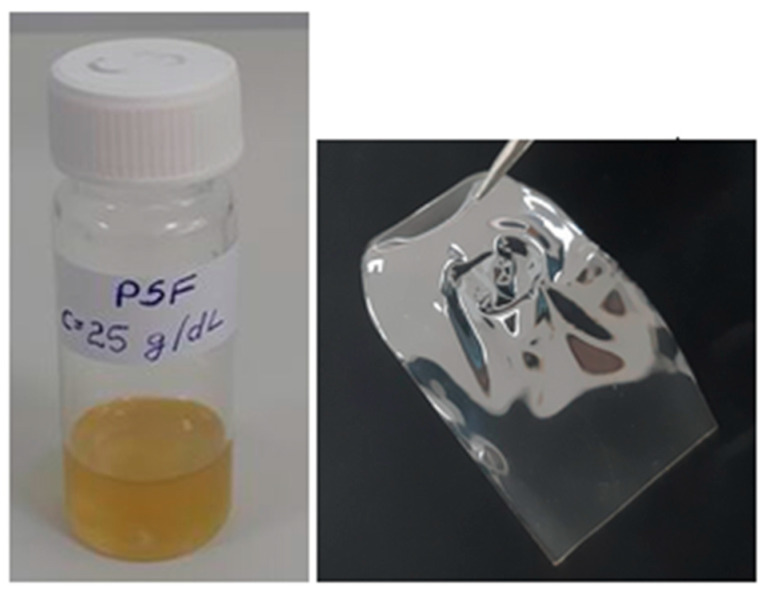
Illustration of the obtained films from the corresponding solutions: exemplified for quaternized polysulfone solution.

The films used in this study (thickness around 40 μm) were obtained by solution-casting method. The process of the obtaining, presented in detail in the previous study [18,26], involves the dissolution of the studied polymers (PSFQ, CAP, PVA) in NMP, obtaining of the homogeneous solutions of 25 g/dL concentration. Moreover, the blends in various ratios were obtained by mixing the two homogeneous solutions (PSFQ with CAP and PSFQ with PVA) by controlling the weight ratio. Finally, the homogeneous solutions thus prepared were cast onto a smooth glass substrate, and solvent has evaporated the appropriate films being formed (illustrated in Figure 12 for the PSFQ solution and film).

#### 3.2.2. Preparation of Fibers by Electrospinning

The polymer homogeneous solutions for electrospinning were prepared as above mentioned. The polymer solutions concentration of 40% was chosen to obtain fibers with sizes in the submicrometer range. The experimental procedure of the processing technique of studied polymers solutions has been previously presented [36] and involves the following parameters: applied voltage potential of 15–20 kV with a needle tip-collector distance of 15 cm and flow rate of the solution through the syringe of 0.75 mL/h. The electrospinning of the PSFQ, CAP and PVA solutions was carried out and continuous fibers were obtained by depositing the fibers randomly over the grounded collector, an aluminum rotary disc.

### 3.3. Methods

#### 3.3.1. Fourier Transform Infrared Spectroscopy (FTIR)

The surface analysis was performed with a Bruker Vertex 70 spectrometer, equipped with a diamond crystal and a single reflexion at incidence angle of 45°, using Attenuated Total Reflection FTIR mode (ATR-FTIR) in the 4000–600 cm^−1^ region.

#### 3.3.2. Rheological Investigations

Rheological measurements were performed using a CS50 Bohlin rheometer with cone-plate geometry (Malvern Instruments, cone angle of 4° and diameter of 40 mm). The viscometric measurements were registered over the 0.05 −3000 s^−1^ shear rate domain, at a temperature of 25 °C. In order to perform the oscillatory measurements in the linear viscoelastic domain (where the storage (G′) and loss (G″) moduli are practically constant and independent on the shear stress), the strain sweep tests were obtained at a frequency of 1 Hz over the strain range of 0.5–10 Pa. Therefore, a shear stress (σ) of 2 Pa was selected, and subsequently, the oscillatory shear measurements were conducted in frequency range of 0.1 and 100 Hz. Rheological tests were obtained with an accuracy of ±5%, for different measurements.

#### 3.3.3. Contact Angle Measurements

The contact angles were measured by the sessile-drop method, with a CAM-101 (KSV Instruments, Helsinki, Finland) contact angle measurement system equipped with a liquid dispenser, video camera, and drop-shape analysis software at room temperature. To evaluate the contact angle at the interface of the polymers surface and water, three different regions of the surface were selected to obtain a statistical result. Thus, the contact angle values of three measurements were taking into consideration with an error of ±1%.

#### 3.3.4. Scanning Electron Microscopy (SEM) and Atomic Force Microscopy (AFM)

The sample morphology was investigated using an environmental scanning electron microscope (ESEM), Quanta 200 operating at an accelerating voltage of 20 kV with secondary electrons in low vacuum mode. AFM imaging was conducted using a Scanning Probe Microscope Solver PRO-M (NTMDT, Zelenograd, Russia) under tapping mode, in air, with a commercially available NSG03 rectangular shaped silicon probe (NTMDT, Zelenograd, Russia). The pores were detected and measured using the Pore Analysis module from Image Analysis 3.5.0.20102 AFM software, using a certain threshold characteristic for every scanned area.

#### 3.3.5. Dynamic Water Vapour Sorption Behavior 

The dynamic water vapors sorption capacity of the films and fibers used in present study have been measured in dynamic regime, using the fully automated gravimetric device IGAsorp system produced by Hiden Analytical (Warrington, UK). The principal part of this equipment is an ultrasensitive microbalance which measures the weight change as the humidity is changed in the sample chamber at a constant temperature. The samples were placed in a special container and dried at 25 °C in flowing nitrogen (250 mL/min.) until the weight of the samples were in equilibrium at a relative humidity, RH, less than 1%, before the sorption/desorption measurements. After that, the RH was gradually increased from 0 to 90%, in 10% humidity steps, every having a preestablished equilibrium time between 30 and 60 min. and the sorption equilibrium was obtained for each step. Then the RH decreased and desorption curves were registered. The system measurements are fully automated and controlled by a software package. 

#### 3.3.6. Antimicrobial Activity Measurements 

The in vitro antimicrobial activity against two bacteria strains, *Staphylococcus aureus*—Gram-positive (*S. aureus*, ATCC 25923) and *Escherichia coli*—Gram-negative (*E. coli*, ATCC 10536), was assessed by the diffusion method (Kirby-Bauer) certified by the National Committee on Clinical Laboratory Standards (NCCLS). Thus, the biological activity of the tested polymers was proved by the occurrence of an inhibition zone whose diameter was measured with a ruler. During antibacterial testing, all measurements were completed in a microbiology laboratory environment of about 24 °C and 55% relative humidity, and repeated four times.

## 4. Conclusions

This study is concentrated on the designing of new materials for target applications. Films and fibers obtained by processing of the quaternized polysulfones, cellulose acetate phthalate, and poly(vinyl alcohol) solutions, as well as their blends (25/75 wt./wt. composition of the PSFQ/CAP and PSFQ/PVA blends) were tested in terms of their bulk/surface properties and performance for targeted bioapplications. According to the surface characteristics of the studied materials, it was observed that an important role is played by the organization form (film or fiber) obtained by processing solution, and also by the features of the added polymers (CAP and/or PVA), as hydrophilic modifiers and pore-forming agents compared with the pure PSFQ substrate. Analyzing the obtained results, we can conclude that both components are well-integrated/stabilized in the structure of the obtained films and fibers and through the superior properties acquired, namely improved flexibility and hydrophilicity, specific molecular microarchitecture, and controlled porosity—properties required by biomedical applications. Studied polymers and blends can be recommended as materials with expected future developments in the biomedical fields. However, an innovative route which offers the possibility of obtaining sustainable materials with the potential to meet the increasing demand in targeted field is represented by development of PSFQ/CAP blends compared to those blends containing PVA as result of the control and improvement of the porosity and hydrophilicity of studied surfaces. All these characteristics have an impact on their performance in the biomedical field, and for this reason there is a great incentive for their use in this domain.

## Figures and Tables

**Table 1:** Surface parameters of the studied materials evaluated based on the sorption/desorption isotherms: water vapor sorption capacity, W, average pore size, $r_{pm}$, and BET data.

Surface	W (%)	$r_{pm}(\mathrm{nm})$	BET Data *	
			Area (m^2^/g)	Monolayer (g/g)
Films				
PSFQ	14.2276	1.35	210.690	0.0600
CAP	9.1037	3.72	48.884	0.0139
PVA	16.9177	9.62	35.185	0.0100
PSFQ/CAP	10.7899	1.98	108.917	0.0310
PSFQ/PVA	26.2046	2.65	197.793	0.0563
Fibers				
PSFQ	23.1467	1.78	260.656	0.0742
CAP	15.4008	1.80	170.516	0.0486
PVA	25.8368	2.98	173.004	0.0493

* Determined based on desorption branch of the isotherm (registered up to a relative humidity of 40%).

## Data Availability

Data supporting the findings of this study are contained within the article and are available from the corresponding author upon request.
